# Exploring Plant–Bacterial Symbiosis for Eco-Friendly Agriculture and Enhanced Resilience

**DOI:** 10.3390/ijms252212198

**Published:** 2024-11-13

**Authors:** Muhammad Qadir, Amjad Iqbal, Anwar Hussain, Adil Hussain, Farooq Shah, Byung-Wook Yun, Bong-Gyu Mun

**Affiliations:** 1 Department of Botany, Abdul Wali Khan University Mardan, Khyber Pakhtunkhwa 23200, Pakistan; qadir1434@gmail.com (M.Q.);; 2 Hunan Key Laboratory of Plant Functional Genomics and Developmental Regulation, College of Biology, Hunan University, Changsha 410082, China; 3 Department of Food Science & Technology, Abdul Wali Khan University Mardan, Khyber Pakhtunkhwa 23200, Pakistan; 4 Department of Entomology, Abdul Wali Khan University Mardan, Khyber Pakhtunkhwa 23200, Pakistan; 5 Department of Agronomy, Abdul Wali Khan University Mardan, Khyber Pakhtunkhwa 23200, Pakistan; 6 Department of Applied Biosciences, Kyungpook National University, Daegu 41566, Republic of Korea; 7 Department of Environmental Biochemistry, Chungbuk National University, Chungbuk 28644, Republic of Korea

**Keywords:** endophytic bacteria, secondary metabolites, plant defense, biofertilizers, biopesticides

## Abstract

This review explores the intricate relationship between plants and bacterial endophytes, revealing their multifaceted roles in promoting plant growth, resilience, and defense mechanisms. By selectively shaping their microbiome, plants harness diverse endophytic bacterial strains to enhance nutrient absorption, regulate hormones, mitigate damage, and contribute to overall plant health. The review underscores the potential of bacterial endophytes in self-sustaining agricultural systems, offering solutions to reduce reliance on fertilizers and pesticides. Additionally, the review highlights the importance of endophytes in enhancing plant tolerance to various environmental stresses, such as drought, salinity, extreme temperatures, and heavy metal toxicity. The review emphasizes the significance of understanding and harnessing the mutualistic relationship between plants and endophytes for maximizing agricultural yields and promoting sustainable farming practices.

## 1. Endophytes

Endophytes are microorganisms that inhabit and operate inside the host’s living tissues. According to Bacon and White [1], these microbes invade and occupy the interior tissues of the host without provoking abrupt negative repercussions or noticeable symptoms. They penetrate the vital, internal parts of plants without manifesting any apparent harmful consequences. These microorganisms (bacteria and fungi) represent a beneficial interaction amongst plants and microbes [1].

### Bacterial Endophytes

Bacterial endophytes are symbiotic bacteria that colonize both intra and intercellular compartments of plants without causing illness or significant morphological changes. The relationship between plants and endophytic bacteria encompasses diverse bacterial taxa and plant species. A defining characteristic of endophytes is their ability to colonize plant tissues without inducing disease, differentiating them from pathogenic microbes [2]. They are found in various plant organs, including roots, stems, leaves, and seeds, and exhibit tissue specificity, with some having a broad host range while others are more specialized. Endophytes are well known for promoting plant growth through mechanisms such as nitrogen fixation, production of phytohormones like indole-3-acetic acid (IAA), and enhancing nutrient availability [3]. They also contribute to stress tolerance, enabling plants to better withstand abiotic stresses like drought, salinity, and heavy metal toxicity through the production of antioxidants and osmoprotectants. Additionally, endophytes can act as biocontrol agents by producing antimicrobial compounds, lytic enzymes, or inducing systemic resistance (ISR) in plants, helping them fend off pathogens. Their diversity and adaptability are further enhanced by horizontal gene transfer (HGT), which enables them to share beneficial traits and adapt to environmental changes. Moreover, endophytes are prolific producers of secondary metabolites, such as siderophores and volatile organic compounds (VOCs), which play roles in nutrient acquisition, plant growth stimulation, and pathogen suppression. These unique characteristics make endophytes crucial components of the plant microbiome, contributing to plant health and resilience and in fact sustainable agricultural practices [4,5].

Soil tillage, irrigation, pesticide usage, and fertilizer application significantly sway the structure and functioning of soil and endophytic microbial consortia. Preserving the ecological variations in plant endophytic bacteria through agricultural approaches is increasingly pivotal for ensuring agricultural output and the superiority of agricultural commodities [6,7]. Different endophytic microbiomes undertake unique and significant roles in maintaining croplands. Plant-inhabiting bacteria have been found to have various positive consequences on plant growth attributes, like facilitating growth, altering metabolic functions, and signaling through phytohormones to adapt to environmental constraints. Utilizing bacterial endophytes in agriculture is particularly relevant for enhancing crop performance amidst circumstances such as cold, drought, polluted soil stress, and providing disease resistance [8,9]. They also hold a pivotal role in the fitness, nutrient assimilation, and stress endurance of the host plant. Bacterial endophytes can be commonly deployed as biopesticides or biofertilizers to counteract biotic challenges (such as pests) and abiotic challenges (for instance, cold, drought, salt, and shifts in soil pH) [10,11]. Abiotic stressors, exacerbated by changing climatic conditions, pose a global problem in achieving optimal crop yields. When plants are subjected to stress, they can activate their endogenous immune systems or overexpress defensive redox regulatory mechanisms to actively scavenge reactive oxygen species (ROS). However, increasing stress factors can exceed plants’ innate redox defense capabilities, inducing extensive internal oxidative impairment and eventual death. Endophytes serve as important internal companions to host plants and have been disclosed to facilitate plants’ response to abiotic stress by modulating site-specific or comprehensive actions against ROS [2,12].

Despite years of research on plant–bacterial interactions, a comprehensive perception of the mechanisms employed by bacteria to enhance plant growth in real-world environments has remained elusive. This lack of knowledge has hindered the consistent utilization of these intricate interactions to boost plant growth effectively. Present understanding suggests that plants can selectively shape their microbiome to host useful bacterial colonizers, involving those residing within their tissues [13,14].

The power of diverse endophytic bacterial strains to support plant development stems from either direct or indirect methods. Direct plant development promotion ensues when a bacterium enhances the absorption of indispensable nutrients or regulates hormones within the host plant species. Plant growth-promoting bacteria (PGPB) can facilitate the accessibility of nutrients such as iron, phosphorus, and nitrogen [15]. Additionally, PGPB can synthesize phytohormones like cytokinin, auxin, and gibberellin to regulate hormone levels. Indirect stimulation of plant development by PGPB transpires when these bacteria mitigate damage caused by phytopathogens, such as certain soil fungi and bacteria. PGPB can hamper the growth of pathogens, which in some ways benefits plant growth [16,17].

## 2. The Ramifications of Agricultural Practices on Soil and Crop Microflora

Land resource management in agriculture significantly influences the biophysiochemical features of soil. For instance, heavy pesticide usage can explicitly impede growth and metabolic functions of microflora and can lead to shifts in microbial populations as agricultural habitats change [18]. Agronomic practices, such as nutrient inputs and outputs, can alter the standard and quantity of plant waste entering the field and its dispersal. The adoption of composted organic matter and inorganic fertilizers has varied impacts on microbial community composition and biomass. Organic manure treatments significantly increase microbial abundance and metabolic functions. Nevertheless, the introduction of fecal microorganisms through manure application can amend the organization of native microbial populations, thus leading to ecological risks [19].

Another dimension of the influence of agricultural practices on soil and crop microflora is the phenomenon of disease-suppressive soil. This refers to soils where hosts are not afflicted by specific ailments or experience reduced ferocity of disease, even when highly pathogenic strains are present in the vicinity of susceptible plants. The activity and soil microbial diversity contribute toward the biological suppression of soilborne diseases [20]. Decades ago, it was discovered that disease-modulating properties of soil were mainly due to enduring monocultures of potato and wheat, which led to the development of species-dedicated microbial populations. Competition for resources, antagonistic interactions through metabolite production, and the promotion of plant systemic defense are some latent mechanisms of disease suppression according to recent research. The impact of bacteria in the genera of *Pseudomonas*, *Streptomyces*, *Bacillus*, and *Actinomyces* has been revealed to contribute significantly to disease suppression in the soil [8].

## 3. A Pathway to Holistic Pest Management and Regenerative Agriculture

A financially sustainable agroecosystem is essential, whether it develops naturally or through human intervention. Farmers often have financial motivations to adopt environmentally friendly agricultural practices. A profitable and productive soil relies on a healthy agroecosystem. It is crucial to explore innovative solutions for constructing sustainable agricultural systems that reduce reliance on agrochemicals as productivity boosters. In the coming decades, plant-associated microorganisms are anticipated to perform an essential role in holistic pest management. Moreover, there is a surging global demand for regenerative agriculture techniques qualified to address rising agricultural production [21]. In this context, endophytes have long been considered a viable alternative approach. The word “endophyte” concerns microbes that establish themselves in plant tissues. The selection of the appropriate plant species, its maturity, and endophytic bacteria are crucial factors in sustainable agriculture. A fundamental understanding of these aspects can support crop production by enabling the use of endophytes as bioinoculants. Analyzing the ecological roles of endophytic bacteria will be pivotal in maximizing agronomic benefits derived from these microorganisms [4,22].

### 3.1. Utilizing Bacterial Endophytes for Sustainable Agriculture

Finding innovative solutions to establish self-sustaining agricultural systems that reduce reliance on fertilizers and pesticides as primary productivity boosters is of utmost importance. Plant-associated beneficial microorganisms are assumed to perform an increasingly vital part in holistic pest control [23]. There is a rising global interest in sustainable agricultural methods that can address the increasing need for agricultural production. These microbes promote plant proliferation through multiple pathways, comprising atmospheric nitrogen fixation (e.g., *Azotobacter*, *Bacillus*, *Clostridium*, and *Klebsiella*), exudation of endogenous and exogenous hormones (IAA, Gibberellic Acid, Salicylic Acid, Abscisic Acid, and Jasmonic Acid), suppression of ethylene synthesis, phosphate solubilization (e.g., *Pseudomonas*, *Bacillus*, *Micrococcus*, *Aspergillus*, and *Fusarium*), and resilience to abiotic stresses (e.g., *Acinetobacter bouvetii*, *Pantoe conspicua,* and *Staphylococcus arlettae*) [24,25]. They can also improve plant mechanisms to counter bacterial pathogens by secreting products like enzymes, siderophores, and antibiotics [18,19]. The selection of appropriate plant species, their maturity, and endophytic bacteria capable of adapting to specific plant tissues are crucial factors in sustainable farming. Having a basic understanding of the mutually beneficial relationship between plants and endophytes can contribute to agricultural production by enabling their use as bioinoculants. A key factor in maximizing agricultural yields from these soil microorganisms would be studying the ecosystems of bacterial endophytes [26].

### 3.2. Bacterial Endophytes as Catalysts for Productive Agri-Food Systems

In the face of multiple limiting factors that hinder agri-food system output, it is crucial to explore avenues beyond these obstacles in a rationalized and agronomically sustainable manner. These endophytes enhance the nutrient acquisition of the host plant such as nitrogen fixation, phosphate solubilization, and reduction of the use of the chemical fertilizer acting as a catalyst to enhance host growth by the improvement in the mechanism of nutrients acquisition. Apart from that, they boost the host defense system to ensure resilience in adverse conditions like pathogen attach, heat stress, chilling stress, and exposure to environmental contaminants [27]. This approach aims to preserve ecological quality while achieving increased productivity through intensified agronomic yields. One viable option that has acquired substantial consideration in recent decades is the utilization of fungal and bacterial endophytes to enhance agricultural output. These endophytes have shown promising potential as phyto-stimulants and as agents for controlling phytopathogens, pests, and insects [28,29].

### 3.3. Bacterial Endophytes as Allies in Plant Growth and Stress Adaptation

Bacterial endophytes extend numerous advantages to their host plants. Bacterial endophytes like *Rhizobium*, *Bradyrhizobium*, *Frankia*, *Pseudomonas,* and *Bacillus* contribute to host proliferation by enhancing nutrient utilization, entailing nitrogen fixation, and yielding plant growth-inducing substances like cytokinin and indole acetic acid (IAA). These endophytes also modulate metabolic reactions and phytohormone signaling, provoking improved endurance in plants to environmental and biological stresses. Bacterial endophytes perform a pivotal role in plant stress adaptation by protecting the host from adverse soil conditions during water scarcity, excessive salinity, and diverse stress situations [30]. For example, the bacterial endophyte *Burkholderia phytofirmans* have been found to boost cold resistance in grapevines by modifying photosynthesis rates and glucose metabolism, crucial for chilling stress tolerance. It helps the host plants adapt to low temperatures by reducing cellular damage, increasing photosynthesis rates, and accumulating cold-responsive molecules, namely proline, starch, and phenolic compounds. Similarly, wheat plants treated with bacterial endophytes like *Priestia aryabhattai* BPR-9 exhibited comparable benefits in metabolic equilibrium and the alleviation of drought stress [31].

*Pseudomonas pseudoalcaligenes*, a bacterial endophyte, has been shown to promote the accumulation of glycine betaine molecules in rice, thus enhancing salinity tolerance [32]. Endophytic *Azospirillum* spp. can surge maize plants’ ability to withstand water stress by accumulating abscisic acid (ABA); this effect is further amplified by the presence of growth-promoters in plants, including gibberellins and IAA. ABA is a plant hormone crucial for various developmental processes, and its levels rise in response to stress. ABA primarily controls the plant hydrological cycle and osmotic stress adaptation [33].

Ethylene, an imperative plant hormone and signaling molecule, is a renowned manager of stress endurance in plants. The enzyme ACC oxidase metabolizes 1-aminocyclopropane-1-carboxylic acid (ACC) to produce S-adenosyl-L-methionine, which ultimately yields ethylene [34]. The build-up of stress-induced ethylene can be detrimental to plant growth and health. Bacteria possess ACC deaminase that enhances the agronomic attributes of the host by metabolizing ACC, the compound that precedes ethylene production. As a consequence, ethylene levels are reduced, and the host’s immune system is stimulated. Given this mechanism, scientists have discovered various bacteria possessing the ability to metabolize ACC through ACC deaminase. Multiple isolates from genera like *Streptomyces*, *Bacillus*, *Isoptericola*, *Serratia*, *Klebsiella*, *Arthrobacter*, *Microbacterium*, and *Pseudomonas* exhibited ACC deaminase activity with attributes that support host growth [35]. Plants pre-exposed to bacterial endophytes capable of deaminase synthesis augmented salinity stress alleviation and increased biomass [35]. Importantly, bacterial endophytes assist the host plant differently from rhizospheric bacteria when subjected to elevated sodium levels. Endophytic bacteria limited sodium levels, while rhizobacteria enabled the host to accumulate salt in the root tissue and potentially compartmentalize it in the vacuolar sap [36]. Isolates of *Commelina communis* with ACC deaminase production enhanced the agronomic characteristics of host plants grown in soils contaminated with lead and zinc from mining activities [8]. During an investigation, the colonization of *Burkholderia phytofirmans* stimulated the metabolism of grapevines under cold stress. This happened through a speedy accrual of gene transcripts and metabolites associated with chilling stress, ultimately contributing to the enhanced tolerance. This discovery sheds light on the priming phenomenon associated with the resistance induced by plant-associated microorganisms. Certainly, the priming of pathogen defense responses is a widely understood process of non-pathogenic plant-associated microorganisms, labeled as induced systemic resistance (ISR) [37]. *Pseudomonas fluorescens* strain 89B-61 was first employed in 1991 to protect cucumber plants against cucumber anthracnose, suggesting that bacterial endophytes may trigger ISR in plants. Bacterial endophytes from the genera *Bacillus*, *Serratia*, and *Pseudomonas* have been proven to stimulate ISR in various phytopathogenic situations, and the cellular signaling responsible for defensive priming has been investigated. ISR enhances the host immune system and protects unexposed plant parts from harmful microorganisms and herbivorous insects. While a salicylic acid-mediated form of ISR has been discovered in various plant-associated bacteria, it is important to note that both ethylene and jasmonic acid also perform a pivotal role in regulating the interconnected signaling pathways necessary for ISR induction [38]. The exact approach of protective priming in ISR is still not fully understood. However, there is proof that supports the involvement of the transcriptional co-repressor NPR1 in JA/ET-dependent ISR. It has been discovered that NPR1 has a function localized in the cytosol in ISR that is distinct from its role in systemic acquired resistance induced by pathogens. Recent findings have shown that the gene expression factors MYB72 and MYC2 contribute to ISR synthesis, induced by rhizobacteria. These gene expression factors are engaged in activating defense genes that are dependent on jasmonic acid and ethylene signaling pathways. Bacterial endophytes present numerous advantages to host plants, including enhanced nutrient acquisition, the synthesis of plant growth-enhancing compounds, the modulation of biochemical reactions and phytohormone signaling, and improved resilience to natural and ecological stresses [39].

### 3.4. Mycorrhizal–Bacterial Interactions

Soil microbiota is essential for sustainable production across various types of agroecosystems. Within the plant microbiota, mycorrhizal fungi (MF) and plant growth-promoting bacteria (PGPB) interact within rhizosphere environments, resulting in additive and/or synergistic effects on plant growth and health [40]. Interactions between mycorrhizal fungi and bacteria support plant growth by enhancing nutrient absorption, lowering ethylene levels, and providing biocontrol against potential pathogens. These benefits occur under both favorable and challenging conditions caused by abiotic or biotic stresses. Such microorganisms are vital to sustainable agriculture, offering growers the potential to reduce or eliminate chemical fertilizers and pesticides. Mycorrhizae, formed by the fusion of fungal mycelium with plant roots, create networks that improve water and nutrient capture from the soil, supporting the plant’s nutrient acquisition [41]. PGPB can increase the availability of nutrients and produce antimicrobial agents that counteract harmful plant pathogens. Certain bacteria also influence mycorrhizal symbiosis with plants; for instance, species from the genus Pseudomonas are well researched for their role in enhancing mycorrhization and are often referred to as Mycorrhiza Helper Bacteria (MHB). The interaction among mycorrhizae, bacteria, and plants presents a valuable approach for sustainable agriculture, especially in areas where abiotic stresses, like soil salinization, limit crop growth and quality [42]. 

Interactions between mycorrhizal fungi and beneficial bacteria are essential in sustainable agriculture, boosting plant health and resilience while reducing the reliance on chemical fertilizers and pesticides. Mycorrhizal fungi, especially arbuscular mycorrhizal fungi (AMF), collaborate with plant growth-promoting endophytic and rhizospheric bacteria to enhance plant growth, particularly under challenging environmental conditions [43]. For instance, inoculating *Zea mays* (maize) with AMF improves phosphorus uptake [44], while introducing *Azospirillum* bacteria supports nitrogen fixation, promoting root development, increased yields, and improved drought resilience [45]. In legumes such as *Glycine max* (soybean), interactions between mycorrhizal fungi and beneficial bacteria can enhance the uptake of essential macro and micronutrients, while also promoting symbiotic nitrogen fixation. This reduces the need for chemical fertilizers, thereby lowering costs and minimizing environmental impact [46]. Recent studies indicate that co-inoculating soybean with *Rhizobium* and AMF increases nodule formation, improves root structure, and enhances grain yield, particularly in soils low in nitrogen and phosphorus. Together, these organisms form a symbiotic relationship that aids the host plant in acquiring essential nutrients [47].

These interactions also play a pivotal role in the ability of disease suppression by the host. For instance, in *Solanum lycopersicum* (tomato), mycorrhizal fungi paired with *Pseudomonas fluorescens* inhibit *Fusarium oxysporum* (a soil born pathogen) infection. *Pseudomonas fluorescens*, categorized as a mycorrhiza helper bacterium (MHB), secrete several biologically active metabolites that trigger mycorrhizal colonization, additionally boosting host defense and their ability to acquire nutrients more efficiently. These bacteria boost root colonization frequency by the mycorrhizal fungi, strengthening the plant’s defense responses via approaches such as Induced Systemic Resistance (ISR) [48]. Apart from that, another such association was recorded in the case of saline soils, where salt-sensitive crops like rice (*Oryza sativa*) have been enabled by such association to endure high saline doses. These interactions enhance the plant’s capacity to expel excess sodium ions (Na⁺) while maintaining osmotic stability by increasing potassium (K⁺) uptake. In the saline soils of Pakistan and India, co-inoculating rice with AMF and *Bacillus* strains has been shown to significantly improve plant growth, allowing farmers to successfully cultivate crops in soils that would otherwise be unproductive [49].

Among the other environmental issues, heavy metals are considered the most pressing issue to the modern world where mycorrhizal–bacterial interactions are invaluable. For instance, in industrial areas, the contamination of heavy metals like cadmium (Cd) and chromium (Cr) and plants like *Helianthus annuus* (sunflower) can buildup and bear higher metal levels when paired with mycorrhizal fungi and metal-resistant PGPB. In one study, inoculating sunflower with AMF and cadmium-tolerant strains of *Pseudomonas putida* reduced Cd uptake in plant tissues by immobilizing the metal within the fungal hyphae and bacterial biofilms, minimizing toxicity [50].

In restoration ecology and forestry, such interactions have been used to develop a resilient plant population in degraded environmental conditions [51]. In forests, *Pinus* and *Eucalyptus* associated with ectomycorrhizal fungi boost their survival rates in such nutrient-poor and drought prone soil conditions, whereas *Frankia,* a nitrogen-fixing bacteria, enhances nitrogen availability. These associations of mycorrhizal–bacterial interactions are employed in the phenomenon of reforestation in the majority of the arid regions of Australia, where co-inoculation with these microbes has enhanced the probability to establish forests and their growth even in nutrient-poor soil conditions [52].

These mycorrhizal–bacterial associations also offer advantages for horticulture. In crops like *Fragaria ananassa* (strawberry), the co-inoculation with AMF and PGPB like *Bacillus amyloliquefaciens* has shown to increase fruit yield, quality, and resistance to fungal phytopathogens. *Bacillus amyloliquefaciens* secrete bioactive antimicrobial metabolites, inhibiting several phytopathogens, while the mycorrhizal fungi on the other hand enhance the host’s ability to absorb nutrients more effectively. Enabling plants naturally to show resistance to pathogens and effectively absorb nutrients from the soil results in more resilient plants with lower pesticide needs, higher resistance, and higher yield qualities and quantities [53].

Remarkably, microbial consortia applications may possibly be tailored to local environments for increased yield. In Mediterranean vineyards, for example, inoculating AMF and *Pseudomonas spp*. to grapevines has verified augmented drought tolerance and nutrient efficacy, predominantly throughout dry growing seasons. These relations have enabled vineyards to sustain grape quality and yield with reduced irrigation, making viticulture more sustainable [54].

In precision agriculture, the tailored use of microbial consortia like AMF and specific PGPB strains can optimize yield and resilience in staple crops. In wheat (*Triticum aestivum*), co-inoculation with AMF and phosphorus-solubilizing bacteria has led to improved phosphorus uptake and boosted grain yield, especially in phosphorus-poor soils common in regions like Sub-Saharan Africa. By utilizing these naturally occurring microbial partnerships, farmers can reduce dependency on synthetic fertilizers, which are often scarce or costly in these regions [55].

### 3.5. Bacterial Endophytes and the Role of Holobiome in Pathogen Protection

**Figure 1:**
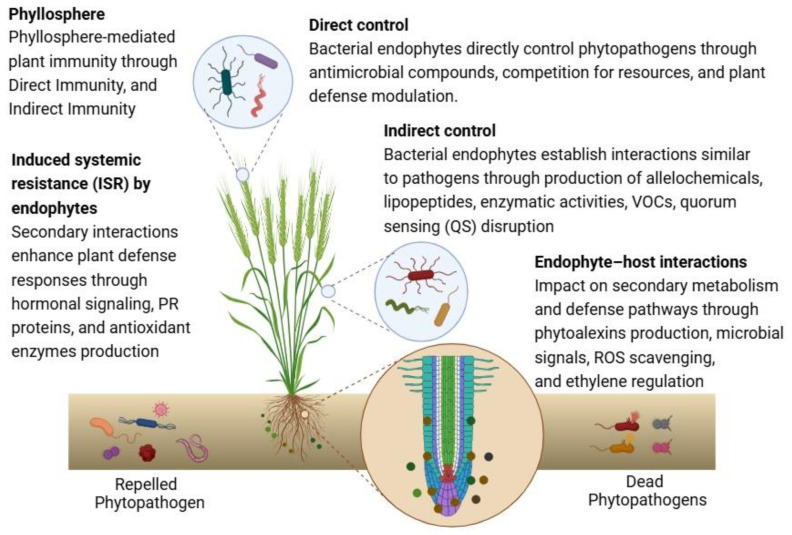
Bacterial endophytes and their role in the holobiome for plant defense. Antimicrobial compounds include enzymes, antibiotics, siderophores, and secondary metabolites; competition for resources means outcompeting pathogens for space and nutrients; plant defense modulation means strengthening plant defense responses; direct immunity means that the holobiome contributes to the extended immunity of the host; indirect immunity means that the holobiome contributes to the enhanced resistance of the host; lipopeptides includes antibacterial compounds, e.g., *Bacillus* and *Paenibacillus*; activities of chitinases, glucanases, proteases degrade pathogens, i.e., disruption of the outer layers of the pathogen’s cell; Volatile Organic Compounds (VOCs) have broad-spectrum antimicrobial action; Quorum Sensing (QS) disruption means inhibiting the pathogenic QS mechanisms; hormonal signaling means the regulation of ethylene, jasmonic acid, and salicylic acid pathways; PR proteins and antioxidant enzymes can enhance plant resilience against phytopathogens; phytoalexins production can be influenced by colonization; antioxidants (flavonoids, phenolics), enzymes (SOD, POD, PPO) help plants deal with ROS; ACC deaminase reduces ethylene signaling. This figure was made using Biorender (https://www.biorender.com/; accessed on 11 August 2024).

The holobiome refers to the integrated ecological entity consisting of a host organism and its associated microbiome, which includes diverse microbial communities such as bacteria, fungi, viruses, archaea, and other microorganisms inhabiting the host’s internal and external environments. To protect the host plant from the phytopathogens in the holobiome, bacterial endophytes employ both direct and indirect mechanisms to shield plants against pathogens (Figure 1). Immediate processes involve the liberation of antimicrobial compounds like hydrolytic enzymes, antibiotics, siderophores, and other secondary metabolites. Indirect pathways include rivalry with pathogens to acquire space and nutrients, as well as the strength to modulate plant defense responses [43].

The benefit of bacterial endophytes has been extensively documented in the literature (Table 1). Lately, a couple of methods have been identified concerning the alteration of microbiome-mediated plant immunity. These approaches are meant to as direct and indirect immunity, signifying two forms of extended immunity credited to the holobiome [44].

## 4. Direct Containment of Phytopathogens by Bacterial Endophytes

The direct containment of phytopathogens can be attained through close interactions and comparable colonization tendencies with host plants [40]. This direct containment of pathogenic growth is primarily accredited to the production of restrictive allelochemicals, including siderophores, antimicrobial agents, cell wall degrading enzymes, volatile organic compounds (VOCs), quinones, alkaloids, terpenoids, steroids, flavonoids, phenolics, or pathogen-quenching signals [80].

Bacterial endophytes possess the capacity to synthesize lipopeptides that belong to a significant group of antibacterial compounds. Among these, *Bacillus* and *Paenibacillus lipopeptides* have been extensively investigated. Within the Bacillus genus, several important lipopeptide producers have been identified, particularly among *Bacillus amyloliquefaciens* isolates [81]. The endosymbiont *Pseudomonas viridiflava* produces ecomycins, a type of lipopeptide that contains uncommon amino acids like β-hydroxy aspartic acid and homoserine [82]. Other antibiotic compounds synthesized by endophytic isolates include polyketides (plipastatin, bacillomycin, iturin, fengycin, surfactin, lichenysin, pumilacidin, and mycosubtilin) generated by *Bacillus subtilis*, as well as polymyxins (a cyclic cationic lipopeptide) manufactured by *Paenibacillus polymyxa* [83,84].

Endophytic lytic enzymes have the proficiency to degrade a wide range of polymers, consisting of cellulose, chitin, lipids, and proteins. Plant-colonizing endophytes exhibit activities involved in the synthesis of enzymes like chitinase, 1,3-glucanase, proteases, and cellulase, which are responsible for hydrolyzing plant cell walls [85]. Chitinase, for example, plays a role in breaking down chitin, the primary component of fungal cell walls. The formation of these enzymes can act as an effective defense mechanism by disrupting the stability of fungal cell walls and jeopardizing their survival. Bacterial endophyte *Streptomyces hygroscopicus*, for instance, produces chitinase that inhibits the proliferation of various fungal phytopathogens such as *Fusarium oxysporum*, *Ralstonia solani*, *Aspergillus niger*, *Alternaria alternata*, *Sclerotinia sclerotiorum*, *Aspergillus flavus*, *Botrytis cinerea*, and *Hyaloperonospora parasitica* [86]. Another study focusing on cotton found that the bacterial endophyte *Bacillus cereus* 65 releases chitinase, which aids the host plant in combating phytopathogens and inhibiting root diseases caused by *Rhizoctonia solani* [87].

Bacterial endophytes produce VOCs that fall into a category of antimicrobial chemicals with a broad spectrum of actions against plant pathogens (fungi, bacteria, and nematodes). Endophytic *Pseudomonas putida* BP25, affiliated with black pepper, has been reported to emit volatile chemicals that suppress the proliferation of fungi, fungi-like species, and plant-infesting nematodes [88]. VOCs have the advantage of facilitating interactions among physically isolated bacteria in addition to their antimicrobial activity. These VOCs can vary in chemical composition. Another direct effect of bacterial endophytes is the reduction of ethylene (ET) levels. ET levels often increase following disease or stress, and various investigations have indicated that inoculating seeds with endophytic bacteria can enhance plant defense. Bacteria can produce 1-aminocyclopropane-1-carboxylate (ACC), which breaks ET into α-ketobutyrate and ammonia. This process decreases the levels of ET, a hormone directly linked to stress and physiological damage in plants [89].

Direct intervention against infections can be achieved by suppressing quorum sensing (QS), a crucial process for the survival of a wide variety of phytopathogens. QS regulates various physiological processes like biofilm formation, reproduction, mutualism, cell-to-cell communication, adaptability, and pathogenicity [90]. Several endophytes have been found to inhibit phytopathogen infection by interfering with quorum sensing through quorum-quenching mechanisms. For example, bacterial endophytes discovered in *Cannabis sativa* L. can derange the cell-to-cell interaction of *Chromobacterium violaceum*. Additionally, strains of *Pseudomonas aeruginosa*, *Rhodococcus corynebacterioides*, and *Stenotrophomonas maltophilia* obtained from the vascular tissue of certain plant species were capable of metabolizing a quorum sensing molecule (3-hydroxy palmitic acid methyl ester) of *Ralstonia solanacearum*. This metabolic activity led to a reduction in bacterial wilt in eggplant [91].

## 5. Unraveling Indirect Interactions and Induced Systemic Resistance by Bacterial Endophytes

Secondary interactions with microbiota contribute to the induction of plant defense responses, specifically via the activation of the immune system to enhance ISR or endophyte-induced tolerance. Different resistance-inducing methods can arise from various encounters or collections of interactions, depending on the specific patho-system [92]. The initial phase of induced defenses (ISR and SAR) is influenced by multiple hormonal signaling networks. ISR is induced by rhizobacteria, bacterial endophytes, and other nonpathogens, while SAR is triggered by infectious agents or chemical compounds. ISR primarily rests on the ethylene and jasmonic acid pathways, achieved through the upregulation of the DEFENSIN 1.2 (PDF1.2) gene. On the other hand, SAR is linked to the upregulation of PR and their corresponding proteins, which are controlled by the salicylic acid-dependent signaling pathway [93]. Recent research has indicated that ISR induced by bacterial as well as other rhizobial strains depends on SA and may also involve JA/ET pathways. Upon inoculation of Pseudomonas fluorescens CHA0 into tobacco roots, the host leaves accumulated PR proteins, which were stimulated by salicylic acid. Additionally, ISR facilitated by the root bacterial endophyte *Micromonospora* against *Botrytis cinerea* banks on the jasmonic acid and ethylene pathways [94].

The initiation of augmented resistance may result from the modification of defense components during endophyte colonization. Interactions between bacteria and their host plants upregulate gene clusters that culminate in the generation of unique metabolites. The association of the host plant with the bacterial cells and the interaction with their metabolites contribute to the capacity of endophytes to enhance plant defenses. Various chemicals produced by endophytes, including lipopeptides, phytohormones, siderophores, pyocyanin, and VOCs, can elicit ISR. Endophytic bacteria that stimulate ISR can shield the host plants from an array of diseases brought about by viruses, nematodes, soilborne pathogenic fungi, biotrophic pathogens, and insect herbivores [95].

## 6. Decoding the Function of Pathogenesis-Related Proteins and Antioxidant Enzymes

Pathogenesis-related proteins (PRs) are renowned for their involvement in adaptive immunity and their activation, which is provoked by necrotic lesions in host plants. Numerous investigations have demonstrated that certain bacterial endophytes can elicit PR activity and enhance resilience to various pathogens. The most extensively studied PR proteins are 1,3-glucanases and chitinases (pertaining to the PR-2 and PR-3 families) [96]. The bacterial endophyte *Bacillus pumilus* SE34 has been found to provoke ISR in host plants, leading to the production of physical and structural barriers, toxins, and 1,3-glucanases that protect the host [92]. Likewise, *B. amyloliquefaciens* TB2 ably controls *Peronophthora litchi* infection in litchi through the synthesis of PR proteins [97]. An actinobacterial endophyte isolated from wheat triggered the increased transcription and translation of PR-1 and PR-4 defense mechanisms in response to *Erwinia carotovora* infection [92]. In maize, *Bacillus* species have been uncovered to fabricate antifungal lipopeptides (fengycin and iturin) and provoke PR genes [98].

Other defensive activities have also been identified to contribute to systemic resistance in several endophytes, including enhanced production of peroxidase (POD), polyphenol oxidase (PPO), superoxide dismutase (SOD), and phenylalanine ammonia-lyase (PAL), among others. When banana seedlings were inoculated with the bacterial endophyte *Serratia marcescens* strain UPM39B3, enzymatic antioxidants such as PPO, peroxidase, PAL, and metabolites like total soluble phenols and lignothioglycolic acid were produced as part of the immune response to prevent Fusarium wilt disease infection [68].

## 7. Impact on Secondary Metabolism and Plant Defense Pathways

The symbiotic association between plants and endophytes can lead to alterations in the biochemical reactions of both the host and the microsymbiont. These alterations may be driven by various processes, considering the influence of endophytes on host defense pathways or the modulation of endophyte metabolism by the plant to limit colonization. Secondary metabolites perform a pivotal role in regulating the interaction of the host with endophytes under various scenarios [99].

Phytoalexins, which are low-molecular-mass antibacterial compounds found in plants, including terpenoids and flavonoids, among others, have been extensively studied for their production in response to pathogen recognition. However, intriguing discoveries have shown that root colonization by mycorrhizal and rhizospheric bacteria significantly influences the composition of essential oils, alkaloids, total phenolics, and terpenoids in the host [100]. Recent investigations have also shown that alterations in anthocyanins, flavonoids, and phenolics were attributed to deferred fungal attack on blackberries subjected to rhizobacterium N17.35. Changes in the levels of various metabolites, including camalexin, phytoalexins, and glucosinolates, have been observed in *Arabidopsis* plants infected with *Pseudomonas fluorescens* SS101 [101].

Microbial signals such as peptides, lipopolysaccharides, and glycoproteins have been established to elicit plant immune responses and the production of metabolites. For example, the lipopeptide fengycin was found to induce the phenylpropanoid pathway metabolism in potato tuber cells. Quorum sensing molecules from other bacterial groups, such as N-acyl-homoserine lactones (AHLs), have also been shown to increase the accretion of oxylipins, phenolic compounds, and salicylic acid in several plant species [91].

Endophytes also perform a role in helping plants cope with reactive oxygen species (ROS) toxicity by governing the production of ROS. They can synthesize various metabolites, including antioxidants, enzymes, and phytohormones, that assist in scavenging ROS. For example, *Festuca rubra*, *Festuca arundinacea*, and *Elymus dahuricus* plants colonized by endophytes showed elevated levels of phenolics and antioxidants [102]. Many bacterial endophytes, such as *Gluconacetobacter diazotrophicus*, *Enterobacter* sp. 638, and *Serratia marcescens* RSC-14, have been found to possess genes encoding ROS-scavenging enzymes [103]. Some endophytes also produce auxins, such as IAA, which can regulate plant cell responses to ROS. However, the task of auxins and other associated chemicals produced by endophytes in plant development, defense, and the regulation of plant–endophyte symbiotic relationships is still not well understood [104].

## 8. Bacterial Endophytes as Enhancers of Plant Resilience to Abiotic Stress

**Figure 2:**
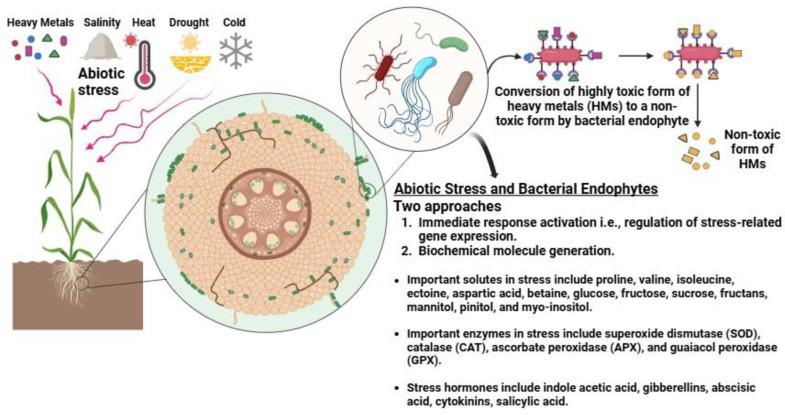
The role of endophytic bacteria in plant growth promotion under abiotic stress. This figure showcases the multifunctional roles of plant growth-promoting (PGP) endophytic bacteria in plant–microbe interactions. PGP endophytic bacteria reside within plant tissues and contribute to plant growth promotion through various mechanisms. They not only produce plant growth promoting chemicals (PGPCs) that enhance nutrient uptake and hormone regulation but also aid in detoxifying both organic and inorganic pollutants present in the environment. Moreover, these bacteria release plant defense chemicals that bolster the plant’s innate defense mechanisms against stresses. This figure was made using Biorender (https://www.biorender.com; accessed on 18 August 2024).

Plants face various environmental pressures and strains imposed by living communities that can limit their growth and development, but bacterial endophytes play a central role in host plant resistance against such stresses (Table 2). Moreover, to tolerate abiotic stress, plants employ two approaches: immediate activation of response systems upon stress exposure, and the generation of biochemical molecules by endophytes with anti-stress properties (Figure 2) [105,106]. The inoculation of host plants with the endophytic *Bacillus* sp., *Arthrobacter* sp. brought about diminished expression of stress-related genes, enhanced plant biomass, nutrient assimilation, growth indices, and eased toxicity under abiotic stress [107]. *Streptomyces padanus* AOK-30 can increase host plant resilience to drought stress through gene regulation. During drought stress, plant species associated with endophytes can produce substantial amounts of free amino acids and sugars to cope with the harsh conditions. Plants colonized by endophytes can achieve high biomass even under conditions of salinity, high or low temperatures, and drought stress. The increased biomass production can be accompanied by higher antioxidant activity, leading to improved seedling development as a response to severe stress [108]. Furthermore, colonization by *B. phytofirmans* enhances CO_2_ assimilation, photosynthesis, water use efficiency, and chlorophyll content in host plants under low water states [109].

Drought, a primary environmental challenge, hampers plant expansion, development, and productivity by limiting water availability to the roots or increasing transpiration rates. Diurnal dehydration is prevalent in most plants during the hours of midday and afternoon in temperate zones, even when soil groundwater levels are sufficient [159]. Short-term drought leads to decreased growth rates, reduced seed viability and vigor, compromised membrane integrity, hindered photosynthetic rates, and increased generation and accumulation of ROS. Osmotic imbalances and ionic stress caused by drought result in premature cell death, while salt stress induces osmotic alterations. The signs of osmotic stress caused by salt, such as stunted growth and leaf senescence, may differ from those of drought stress in the shoot system [109]. Plants hosting endophytes, such as dune grass, panic grass, tomato, and rice, exhibit enhanced drought tolerance characterized by reduced water requirements and increased biomass production. This improvement can be attributed to several factors, including increased solute accumulation in host tissues colonized by endophytes, thicker cuticles, and decreased leaf transpiration and conduction. Drought tolerance is associated with morphophysiological and genomic adaptations, as well as physiological responses. The primary response to water scarcity involves increased abscisic acid (ABA) production or decreased ABA metabolism. ABA is proposed to function as a signaling molecule in drought-stressed plants, regulating their tolerance to water scarcity by reducing water loss and stomatal closure. Another hypothesis suggests that ABA stimulates root branching, thereby enhancing the plant’s capacity for water absorption, as observed in host plant species associated with *Azospirillum brasilense* Sp 245 [160].

Soluble salt accretion in the soil leads to salinity stress, impacting ecological quality, agricultural productivity, and the economy. Initially, saline conditions negatively affect the metabolic activities of soil microflora, leading to reduced soil output. In later stages, salinity can result in the death of flora and other soil inhabitants, transforming the climax community into barren and desertified land. A soil is categorized as saline when the electrical conductivity of the extract obtained at saturation in the root zone exceeds 4 dS m^−1^ at 25 °C and contains approximately 15% exchangeable sodium (approximately 40 mM NaCl) [161]. Most agricultural plants exhibit reduced yields at elevated electrical conductivity (ECe) levels, and many crops experience decreased productivity even below the threshold levels of ECe. This phenomenon significantly contributes to the declining productivity of farmed soils [162]. Although precise measurements are challenging, the area of saline soils is expanding, particularly in irrigated regions. Saline conditions currently impact more than 20% of arable land, and it is projected that by 2050, this will increase to nearly 50% of vital agricultural land. Endophytes can enhance agronomic qualities, modify metabolism, and facilitate phytohormone signaling. These microorganisms also play a pivotal part in the adaptation of plants to salinity and other stresses. Endophytic microbes are particularly advantageous for improving crop stress tolerance as they are somewhat shielded from the stark soil environment, enabling them to effectively cope with challenges like salinity [152,163].

Extreme temperatures have detrimental effects on plant development, with high temperatures causing conformational changes in cellular proteins and low temperatures inhibiting metabolism by disrupting enzyme activities, intermolecular interactions, protein structure, and membrane characteristics. The negative impacts of severe temperatures are often associated with water scarcity [164]. *B. phytofirmans* has been shown to enhance the resilience of host plants exposed to temperatures below threshold levels [109]. The grass *Dichanthelium lanuginosum* is capable of thriving in Yellowstone National Park, where temperature fluctuations range from 38 °C to 65 °C [165]. *Curvularia protuberata*, along with its heat-resistant mycovirus *Curvularia* (CThTV), can tolerate stress and promote host growth under high temperatures [166]. Wheat yield and germination were improved in the second generation due to microbial endophytes. The presence and distribution of plant endophytes can be affected by various environmental factors, such as temperature, precipitation, and latitude. In regions characterized by high precipitation and annual temperature, sweet root (*Osmorhiza depauperata*) tends to harbor *Agrobacterium tumefaciens* and *Sinorhizobium meliloti* as more common endophytes. Conversely, in areas with lower rainfall and higher latitudes, *Paenibacillus* strains are found to be more prevalent as endophytes in sweet root. Endophyte-colonized hosts exhibit higher tolerance to thermal and salinity stress, which is attributed to variations in the redox forms of glutathione and ascorbate, as well as reduced peroxidation. Endophytes augment the proficiency of plants to acclimate to chilling conditions by reducing cellular injury, enhancing photosynthesis, and accumulating phenolic compounds, proline, and starch, among other substances associated with chilling stress [167].

Heavy metal toxicity is a significant abiotic stressor that can cause a loss of 25–80 percent in diverse crop yields. Toxicity from metals such as manganese and aluminum, as well as mineral deficiencies in potassium, magnesium, phosphorus, and calcium, are the primary factors contributing to decreased agricultural productivity and reduced soil fertility in acidic soils. Metal contaminants pose a particular threat to the roots of cultivated plants, leading to poor root development. The accretion of heavy metals and their toxic effects in acidic soils has turned into a major concern, limiting crop yields and affecting various physicochemical processes such as nutrient metabolism, photosynthetic and respiratory rates. Bacterial endophytes can perform a task in the mobilization and immobilization of metal cations, thereby influencing the accessibility of cations for plants [168,169]. In soil contaminated with cadmium (Cd), *Exophiala pisciphila*, a dark septate endophyte associated with *Zea mays* roots, demonstrated increased antioxidant enzyme activity [170]. When plants infected with dark septate endophytes were exposed to high levels of Cd (cadmium), three crucial genes responsible for Cd detoxification, transport, and absorption exhibited altered expression patterns. Specifically, PCS (phytochelatin synthase) and MTP (metal tolerance protein) were found to be overexpressed, while ZIP (Zrt- and Irt-like protein) was downregulated. Additionally, *Gigaspora* and *Pseudomonas* can directly modify the concentration of ACC, leading to alterations in heavy metal resilience in plants [166].

## 9. Conclusions

In conclusion, this study investigates the intricate symbiosis between plants and bacterial endophytes, shedding light on their diverse and crucial roles in promoting plant growth, resilience, and defense mechanisms. PGPB emerges as a base in the field of sustainable agriculture, where they facilitate nutrient uptake, synthesize phytohormones, and enhance overall plant health. The review underscores the potential of bacterial endophytes as key players in establishing self-sustaining agricultural systems, offering promising solutions to lessen dependence on fertilizers and pesticides. Moreover, the review highlights the critical role of endophytes in fortifying plant resilience against a spectrum of environmental stressors, including drought, salinity, extreme temperatures, and heavy metal toxicity. This emphasizes the importance of comprehending and harnessing the mutualistic bond between plants and endophytes, not only for optimizing agricultural yields, but also for advancing sustainable farming practices. In doing so, this review contributes valuable insights that may pave the way for more resilient, eco-friendly, and productive agricultural systems in the future.

## Figures and Tables

**Table 1:** A list of bacterial endophytes that help host plants under biotic stress.

S. No.	Bacterial Endophyte	Role in Pathogen Protection	References
1	*Achromobacter* spp.	Enhances nutrient uptake, induces systemic resistance	[45]
2	*Acinetobacter* spp.	Promotes plant growth, reduces pathogen infection	[46]
3	*Actinobacteria* spp.	Diverse roles, antibiotics production, and plant growth promotion	[47]
4	*Aeromonas* spp.	Enhances stress tolerance, reduces pathogen infection	[48]
5	*Agrobacterium tumefaciens*	Causes crown gall disease but can be used in genetic engineering	[49]
6	*Arthrobacter* spp.	Produces antifungal and antibacterial metabolites	[50]
7	*Azospirillum* spp.	Enhances root growth, improves nutrient uptake	[51]
8	*Bacillus pumilus*	Produces antimicrobial compounds, improves stress tolerance	[52]
9	*Bacillus subtilis*	Produces antibiotics, induces systemic resistance	[53]
10	*Bradyrhizobium* spp.	Nitrogen fixation influences plant health	[54]
11	*Burkholderia* spp.	Produces antifungal metabolites, enhances root growth	[55]
12	*Caulobacter crescentus*	Improves nutrient uptake, pathogen protection	[56]
13	*Citrobacter* spp.	Enhances nutrient availability, may reduce pathogen infection	[57]
14	*Desulfovibrio* spp.	Role in pathogen protection not well understood	[58]
15	*Enterobacter cloacae*	Enhances plant growth, may inhibit pathogens	[59]
16	*Erwinia* spp.	Causes disease but some strains can trigger plant defenses	[60]
17	*Eubacterium* spp.	May influence plant health indirectly	[61]
18	*Firmicutes* spp.	Nitrogen fixation and production of antimicrobial compounds	[62]
19	*Flavobacterium* spp.	Produces antimicrobial compounds, enhances stress tolerance	[63]
20	*Klebsiella* spp.	Nitrogen fixation, enhances plant growth, may inhibit pathogens	[64]
21	*Mesorhizobium* spp.	Nitrogen fixation influences plant health	[65]
22	*Methylobacterium* spp.	Enhances stress tolerance, reduces pathogen infection	[66]
23	*Microbacterium* spp.	Induces systemic resistance, produces antimicrobial compounds	[67]
24	*Mycobacterium* spp.	Some strains may induce plant defense mechanisms	[68]
25	*Paenibacillus* spp.	Induces systemic resistance, produces antimicrobial compounds	[69]
26	*Pantoea agglomerans*	Triggers plant defenses, reduces pathogen infection	[70]
27	*Pseudomonas fluorescens*	Antifungal compounds, induces plant defense mechanisms	[71]
28	*Pseudonocardia* spp.	Some strains may protect plants from pathogens	[72]
29	*Ralstonia* spp.	Some strains can induce plant resistance	[73]
30	*Rhizobium* spp.	Nitrogen fixation, induces plant defense responses	[74]
31	*Serratia marcescens*	Produces antifungal and antibacterial compounds	[75]
32	*Stenotrophomonas maltophilia*	Induces systemic resistance, improves nutrient uptake	[76]
33	*Streptomyces* spp.	Produces antibiotics, induces plant resistance	[77]
34	*Variovorax paradoxus*	Enhances plant growth, may compete with pathogens	[78]
35	*Xanthomonas citri*	Induces systemic resistance against certain pathogens	[79]

**Table 2:** A list of bacterial endophytes that can alleviate abiotic stress in hosts.

S. No.	Bacterial Endophyte	Abiotic Stress	References
1	*Achromobacter* spp.	Osmotic stress, heavy metals	[110,111]
2	*Achromobacter xylosoxidans*	Drought tolerance, osmotic stress	[112]
3	*Actinobacteria* spp.	Drought tolerance, secondary metabolite production	[113]
4	*Agrobacterium* spp.	Genetic modification, stress resistance	[114]
5	*Alcaligenes* spp.	Metal tolerance, stress resistance	[115]
6	*Aminobacter aminovorans*	Drought tolerance, metal tolerance	[116]
7	*Aminobacter* spp.	Drought tolerance, metal tolerance	[116,117]
8	*Azospirillum* spp.	Nitrogen fixation, drought resistance	[118]
9	*Bacillus altitudinis*	Drought tolerance, growth promotion	[119]
10	*Bacillus* spp.	Drought resistance, growth promotion	[120]
11	*Brevundimonas* spp.	Osmotic stress, growth promotion	[121]
12	*Chitinophaga* spp.	Metal tolerance, organic matter degradation	[122]
13	*Chryseobacterium* spp.	Drought tolerance, metal accumulation	[123,124]
14	*Curtobacterium* spp.	Drought tolerance, cold resistance	[125,126]
15	*Dyella* spp.	Metal tolerance, stress resistance	[127]
16	*Enterobacter cloacae*	Drought tolerance, metal detoxification	[128]
17	*Erwinia* spp.	Drought resistance, plant growth promotion	[129]
18	*Flavobacterium* spp.	Salinity, metal tolerance	[130]
19	*Klebsiella oxytoca*	Drought resistance, salinity tolerance	[131]
20	*Kocuria* spp.	Drought tolerance, growth promotion	[132]
21	*Lysinibacillus* spp.	Drought tolerance, growth promotion	[133]
22	*Marinobacter* spp.	Salinity tolerance, osmotic stress	[134]
23	*Massilia* spp.	Metal tolerance, stress resistance	[135]
24	*Methylobacillus* spp.	Cold, heat tolerance, growth promotion	[136]
25	*Methylobacterium extorquens*	Drought, salt tolerance, stress resistance	[137]
26	*Methylobacterium* spp.	Cold tolerance, stress protection	[138]
27	*Microbacterium* spp.	Drought tolerance, plant growth promotion	[139]
28	*Mitsuaria chitosanitabida*	Cold tolerance, plant growth promotion	[140]
29	*Novosphingobium* spp.	Drought tolerance, metal resistance	[141,142]
30	*Ochrobactrum* spp.	Metal tolerance, plant growth promotion	[143]
31	*Ochrobactrum* spp.	Drought tolerance, metal tolerance	[104,143]
32	*Paenibacillus* spp.	Drought tolerance, nutrient solubilization	[144,145]
33	*Pantoea agglomerans*	Heat, cold, drought resistance	[144,145,146,147]
34	*Pseudomonas putida*	Salinity, heavy metals, osmotic stress	[148]
35	*Pseudomonas* spp.	Drought, salinity, heavy metals	[149]
36	*Rhizobium* spp.	Drought, salinity, nutrient stress	[145,150]
37	*Rhodococcus* spp.	Drought tolerance, biodegradation	[151]
38	*Serratia marcescens*	Drought resistance, heavy metals	[152,153]
39	*Streptococcus* spp.	Cold, heat tolerance, stress resistance	[154]
40	*Streptomyces* spp.	Drought, heat, salinity resistance	[155,156]
41	*Variovorax* spp.	Drought tolerance, biodegradation	[125]
42	*Weissella* spp.	Heat, cold tolerance, plant growth promotion	[157]
43	*Xanthomonadaceae*	Abiotic stress mitigation, biocontrol	[158]

## Data Availability

All the relevant data are available within the manuscript. Any additional information can be provided upon request to the corresponding author.
